# Nailfold capillary abnormalities in childhood-onset systemic lupus erythematosus: a cross-sectional study compared with healthy controls

**DOI:** 10.1177/0961203321998750

**Published:** 2021-03-03

**Authors:** Dieneke Schonenberg-Meinema, Sandy C Bergkamp, Amara Nassar-Sheikh Rashid, Leontien B van der Aa, Godelieve J de Bree, Rebecca ten Cate, Maurizio Cutolo, A Elisabeth Hak, Petra CE Hissink Muller, Marieke van Onna, Taco W Kuijpers, Vanessa Smith, J Merlijn van den Berg

**Affiliations:** 1 Department of Pediatric Immunology, Rheumatology and Infectious Diseases, Emma Children’s Hospital, Amsterdam University Medical Centers (Amsterdam UMC), University of Amsterdam, Amsterdam, the Netherlands; 2 Department of Pediatric Rheumatology, Leiden University Medical Centre (LUMC), Leiden, the Netherlands; 3 Department of Infectious Diseases, Amsterdam University Medical Centers (Amsterdam UMC), University of Amsterdam, the Netherlands; 4 Department of Internal Medicine, Research Laboratory and Academic Division of Clinical Rheumatology, IRCCS Polyclinic San Martino Hospital, University of Genova, Genova, Italy; 5 Department of Rheumatology and Clinical Immunology, Amsterdam Rheumatology and Immunology Centre, Amsterdam University Medical Centers (Amsterdam UMC), University of Amsterdam, Amsterdam, the Netherlands; 6 Department of Rheumatology, Ghent University Hospital, Belgium; 7 Faculty of Internal Medicine, Ghent University, Ghent, Belgium; 8 Unit for Molecular Immunology and Inflammation, VIB Inflammation Research Centre (IRC), Ghent, Belgium

**Keywords:** Capillaroscopy, systemic lupus erythematosus, pediatric, childhoodonset, case-control

## Abstract

**Objectives:**

For selection of high-risk systemic lupus erythematosus (SLE) patients it is necessary to obtain indicators of disease severity that predict disease damage. As in systemic sclerosis, nailfold capillary abnormalities could be such a biomarker in SLE. The primary objective of this cross-sectional study is to describe capillary abnormalities in childhood-onset SLE (cSLE) cohort (onset < 18 years) and compare them with matched healthy controls. The secondary objective is to correlate the observed capillary abnormalities with demographical variables in both cohorts and with disease-specific variables in cSLE patients.

**Methods:**

Healthy controls were matched for ethnic background, age and gender. Videocapillaroscopy was performed in eight fingers with 2-4 images per finger. Quantitative and qualitative assessments of nailfold capillaroscopy images were performed according to the definitions of the EULAR study group on microcirculation in Rheumatic Diseases.

**Results:**

Both groups (n = 41 cSLE-patients and n = 41 healthy controls) were comparable for ethnic background (p = 0.317). Counted per mm, cSLE-patients showed significantly more ‘giants’ (p = 0.032), ‘abnormal capillary shapes’ (p = 0.003), ‘large capillary hemorrhages’ (p < 0.001) and ‘pericapillary extravasations’ (p < 0.001). Combined ‘abnormal capillary shapes and pericapillary extravasations’ (in the same finger) were detected in 78% (32/41 patients). By qualitative analysis, ‘microangiopathy’ was detected in 68.3% (28/41) and a ‘scleroderma pattern’ in 17.1% (7/41) of the cSLE-patients (without scleroderma symptoms). The difference of percentage positive anti-RNP antibodies in the group with or without a scleroderma pattern was not significant (p = 0.089). The number of ‘abnormal capillary shapes per mm’ was significantly correlated with treatment-naivety. The number of ‘large pathological hemorrhages per mm’ was significantly correlated with SLEDAI score and presence of nephritis. Compared to healthy controls, ‘pericapillary extravasations’ were found in significantly higher numbers per mm (p < 0.001) as well as in percentage of patients (p < 0.001).

**Conclusions:**

Our observations confirm that giants, abnormal capillary morphology and capillary hemorrhages are also observed in cSLE, as was already known for adults with SLE. Number of capillary hemorrhages in cSLE was significantly correlated with disease activity. A high frequency and total amount of “pericapillary extravasations” was observed in cSLE patients, possibly revealing a new subtype of capillary hemorrhage that might reflect endothelial damage in these pediatric patients.

## Introduction

Nailfold capillaroscopy (NFC), a non-invasive magnification method, is used to visualize the capillaries of the fingertips. NFC is a diagnostic instrument, used in patients with Raynaud’s phenomenon: a capillary scleroderma pattern is associated with systemic sclerosis (SSc).^1–3^

Systemic Lupus Erythematosus (SLE) patients can also show capillary abnormalities in NFC. As concluded in a recent review, adults with SLE show a significantly higher number of tortuous capillaries, abnormal capillary morphology, hemorrhages and “semi-quantitative NFC score”, when compared to healthy controls.^4^ Additionally, the NFC-score (by rating severity of capillary changes) also seems to correlate with disease activity.^4^ Studies on nailfold capillary findings in children with SLE are scarce and inconclusive. In our recently published systematic review, data from six published studies on this topic were not comparable as different definitions for abnormal morphology were used.^5^ Moreover, the definition for abnormal capillary morphology was recently further specified and revised by the European League Against Rheumatism (EULAR) Study Group on Microcirculation in Rheumatic Diseases (SG MCRD).^6,7^

The diagnosis of childhood-onset (c)SLE is often delayed due to heterogeneity of presenting symptoms, and is dependent on recognition by and experience of the clinical physician. To prevent organ damage, it is important to prevent delay in diagnosis. Delay in diagnosis is specifically mentioned as one of the patients’ unmet needs in a recent publication of ‘state of the art on clinical guidelines’.^8^ Prevention of delay in diagnosis is especially important for cSLE-patients, because it was shown that they have more severe symptoms at presentation and a more severe disease course compared to patients with adult onset SLE.^9–13^ Heterogeneity is not only applicable for disease symptoms but also for disease severity with mild to severely affected patients and, depending on type of organ manifestations, a higher risk of mortality.^12^ SLE is associated with progressive (irreversible) organ damage, which has shown to be a predictor of additional morbidity and early mortality.^14^ A recent international recommendation for treatment in SLE is based on the treat-to-target principle: ‘since damage predicts subsequent damage and death, prevention of damage accrual should be a major therapeutic goal in SLE’.^15^ Steroid-related damage is an important factor in SLE and has become an outcome parameter for damage in long-term SLE follow-up studies.^16,17^ Selection of patients who need aggressive and steroid-sparing treatment in early phases of the disease will lead to less organ damage and lower cumulative steroid-use. For selection of high-risk patients it is necessary to obtain indicators of disease severity that predict (severe) future disease damage. Nailfold capillary abnormalities could be such an indicator or biomarker in SLE. For systemic sclerosis (SSc), multiple studies have shown that capillary abnormalities (by qualitative description) can be of use as a prognostic biomarker.^18–22^

This study was conducted by the EULAR SG MCRD. The primary objective of this cross-sectional study is to describe possible capillary abnormalities in cSLE patients and compare them with healthy controls, matched for skin pigmentation, age and gender. These demographic variables have been described as confounding factors in healthy controls in interpreting capillary characteristics, such as density.^23,24^ The secondary objective is to correlate the observed capillary abnormalities with demographical variables in both cohorts and with disease-specific variables in cSLE patients.

**Figure 1.**
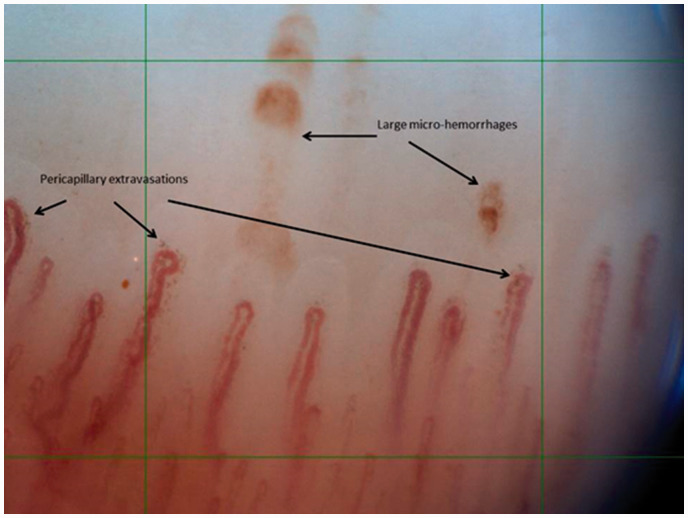
‘Large hemorrhages’ versus ‘pericapillary extravasations’.

**Figure 2.**
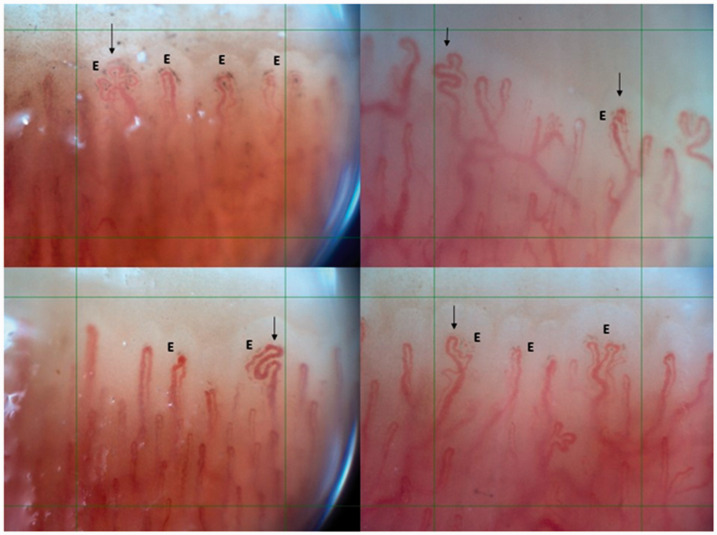
Combination of pericapillary extravasations (E) and abnormal shapes (black arrows).

## Patients and methods

### Patients and controls

Consecutive patients with (suspected) cSLE were cross-sectional included during a visit at the (outpatient) clinic. Criteria for inclusion were SLE diagnosis by using the 2012 classification criteria according to Systemic Lupus International Collaborating Clinics (SLICC)^25^ and age of disease onset < 18 years old. Patients were excluded if they did not fulfil a minimum of four SLICC criteria, if they declined capillaroscopy examination/analysis of their capillaroscopy images, if it was impossible to collect images with good quality (due to nailfold skin thickness) or when a patient was too sick to undergo capillaroscopy examination. Demographical and clinical data were collected from patient charts. For cSLE-patients with one-time cross-sectional capillaroscopy, informed consent was waived by our ethical committee. Nevertheless, most cSLE-patients were part of a longitudinal cohort study for which an informed consent by patients (from 12 years of age) and/or both parents (for patients below 16 years) was signed. If capillaroscopy was performed longitudinally, images from the first capillaroscopy were used.

For healthy controls, children and adolescents from schools around the Amsterdam University Medical Centers (AUMC) and via personal contacts of the authors were approached for one-time capillaroscopy. This project was approved, combined with our longitudinal cSLE cohort study (Dutch trial register registration no. NL60885.018.17) by the ethical committee from the AUMC. Inclusion of healthy children followed if they did not suffer from a chronic disease and had signed informed consent (child from 12 years of age and/or both parents for children below 16 years old). Age, gender, ethnic background, Raynaud symptoms and periungual trauma were noted. Disease activity in cSLE patients was measured by Systemic Lupus Erythematosus Activity Index (SLEDAI) score. Patients and healthy controls were coded with an unique study number.

### Nailfold capillaroscopy technique and image collection

NVC was performed with a x200 magnification lens from Optilia. All images were collected by one investigator (DS). The patients/healthy children stayed in a room of 20-22°C for a minimum of 15-20 minutes. During capillaroscopy they were in sitting position with the hand on a table at the level of their heart. A drop of oil was applied to the fingers before examination. In total, eight fingers per cSLE-patient (excluding the thumbs) were examined. Per finger, four images were stored. From November 2017 until June 2018, a cohort of healthy children was included. In healthy children, eight fingers were examined and two images per finger were stored (according to the EULAR SG MRCD study protocol). From this larger healthy pediatric study cohort, healthy controls were matched with our cohort of cSLE-patients according to ethnic background, age and gender (in that order).

### Image analysis

Post-examination, the following quantitative capillaroscopy characteristics were evaluated by primary investigator (DS) with a grid per millimeter: density (number of capillaries in distal row per mm), number of abnormal shapes (as defined by EULAR SG MCRD as all other shapes than hairpin (stereotype hairpin shape), crossing (once or twice) and tortuous (limbs bend but do not cross)),^6,7^ number of giant capillaries (if apical diameter >50 µm), maximum apical diameter (in µm, by Optipix software version 1.7.6), and number of capillary hemorrhages.^3^ Hemorrhages were defined in two subtypes: ‘large pathological hemorrhages’ as large deposit of hemosiderin with a cap-like appearance^1^ and ‘pericapillary extravasations’ as small point-shaped hemorrhages surrounding the capillary loop (Figure 1). Examined subjects were asked for finger trauma and manicure treatment in the 2-3 weeks prior to examination.

Qualitatively, three capillary patterns were described. A scleroderma pattern was defined by presence of giant capillaries, possibly combined with large pathological capillary hemorrhages, loss of capillaries and abnormal capillary shapes, according to the ‘Fast Track Algorithm’.^26^ If the observed capillary pattern showed abnormal capillary morphology or hemorrhages, but did not match the criteria for scleroderma, it was called ‘microangiopathy’, referring to non-specific abnormalities. A normal pattern showed no capillary abnormalities.

Capillaries from images of low visible quality were excluded and not analyzed.

### Statistical analysis

Statistical analysis was performed with IBM SPSS Statistics type 26. Descriptive statistics were reported in terms of percentages, means and standard deviations or medians and inter-quartile ranges depending on distribution of outcome data. Demographical differences between both study groups were calculated with a paired t-test (in case of normal data distribution), McNemar test (for binary and nominal outcome variables) and Wilcoxon signed rank test (in case of no normal data distribution). Linear regression by ANOVA and logistic regression were used for respectively numerical and categorical outcome data. Demographic and clinical variables (only for the cSLE-cohort) were tested as co-variate factors for the amount (per mm) of ‘abnormal capillary shapes’, ‘large hemorrhages’ or ‘pericapillary extravasations’. Type of ethnic background was analyzed as an ordinal variable for three types of skin pigmentation: white/white-mixed, Asian/North-African/Middle-eastern and African/Afro-Caribbean. P-values <0.05 were considered as statistically significant.

## Results

### Inclusion and demographics

Fifty-two patients with (suspected) cSLE were eligible for inclusion between April 2016 until September 2019. After revising SLICC-criteria, seven patients did not fulfil a minimum of four criteria and were excluded. Two patients were excluded because it was not possible to obtain clear capillaroscopy images due to skin thickness around their nailfolds. One patient, with circulatory insufficiency admitted on intensive care unit, was too sick to undergo capillaroscopy examination. Therefore, forty-one patients were included for analysis.

The same number of healthy controls (n = 41) were matched from a cohort of healthy children (n = 140) with capillaroscopy images, first by matching for ethnic background (p = 0.317). The cSLE-cohort had significantly more female patients (36 (87.8%) versus 29 (70.7%), p = 0.039) and higher median age (median 17 versus 12 years, p < 0.001) compared to healthy controls (see Table 1).

**Table 1.** Demographical variables and clinical characteristics of study groups.

	cSLE-patients, n = 41	Healthy controls, n = 41	p-Value
Female, n (%)	36 (87.8)	29 (70.7)	**0.039**
Ethnicity, n (%)			0.317
African/Afro-Caribbean	18 (43.9)	15 (36.6)	
White	15 (36.6)	14 (36.6)	
North-African/Middle-Eastern	3 (7.3)	4 (9.8)	
Asian	3 (7.3)	5 (12.2)	
Mixed/other	2 (4.9)	2 (4.9)	
Age at capillaroscopy in years, median (IQR)	17 (14–18)	12 (11–16.5)	**<0.001**
Raynaud’s phenomenon / acro-cyanotic symptoms, n (%)	14 (34.1)	2 (4.9)	**0.002**
Age at onset in years, median (IQR 25-75)	14 (12.5–16)		
Disease duration in months, median (IQR)	12.9 (0.1–44.5)		
Prednisone naive, n (%)	23 (56.1)		
ANA at diagnosis, n (%)	41 (100)		
ANA + anti-ds-DNA	26 (63.4)		
ANA + anti-RNP	16 (39)		
ANA + anti-Sm	14 (34.1)		
Cutaneous involvement, n (%)	27 (65.9)		
Nephritis, n (%)	13 (31.7)		
Neuropsychiatric involvement, n (%)	6 (14.6)		
Antiphospholipid antibodies, n (%)	5 (12.2)		
SLEDAI score at diagnosis, median (IQR)	12 (8–16)		
SLEDAI score at capillaroscopy, median (IQR)	5 (3–10.5)		

Bold indicates statistically significant p values (<0.05).

^a^McNemar test ^b^Wilcoxon signed rank test: ordinal variables (3 groups: white/mixed/other, Asian/North-African/Middle-Eastern and African/Afro-Caribbean) ^c^paired t-test.

ANA: Anti-Nuclear Antibodies; anti-ds-DNA: anti-double stranded DNA antibodies; anti-RNP: anti-Ribonucleoprotein; anti-Sm: anti-Smith antibodies; SLEDAI: Systemic Lupus Erythematosus Disease Activity Index; IQR: interquartile range.

In total, 8055 capillaries from 1147 images could be analyzed from 41 cSLE-patients. From healthy controls (n = 41), 4253 capillaries were analyzed from 656 images. Disease characteristics of the cSLE-cohort are shown in Table 1. Fifty six percent (56.1%) of patients were treatment naive and investigated at time of diagnosis.

### Quantitative capillary variables

Compared to healthy controls and counted per millimeter, cSLE-patients showed significantly more giant capillaries (p = 0.032), abnormal capillary shapes (p = 0.03), more large pathological hemorrhages (p < 0.001) and more pericapillary extravasations (p < 0.001) (Table 2). In total, large pathological hemorrhages and pericapillary extravasations were significantly more observed in respectively 75.6% (31/41) and 87.8% (36/41) of cSLE-patients compared to healthy controls (resp. in 17.1% (7/41) and in 36.6% (15/41), McNemar test; resp. p < 0.001 and p < 0.001)).

**Table 2.** Capillary characteristics.

Quantitative parameters	cSLE-patients, n = 41	Healthy controls, n = 41	p-Value
Density per mm, mean (SD)	6.83 (1.06)	6.53 (0.86)	0.117
Max apical diameter in µm, median (IQR)	37.7 (35.2–45.9)	38.6 (32.8–41.2)	0.206
Giant capillaries per mm, mean (SD)	0.04 (0.13)	0.003 (0.013)	**0.032**
Abnormal shapes per mm, median (IQR)	0.31 (0.13–0.73)	0.21 (0.06–0.38)	**0.003**
Hemorrhages per mm, median (IQR)	1.1 (0.39–2.34)	0 (0–0.16)	** **
Large pathological hemorrhages per mm	0.07 (0–0.24)	0 (0–0)	**<0.001**
Pericapillary extravasations per mm	1.11 (0.28–2.15)	0 (0–0.13)	**<0.001**
**Qualitative patterns**			
Normal capillary pattern, n (%)	6 (14.6)	37 (90.2)	**<0.001**
Microangiopathy, n (%)	28 (68.3)	4 (9.8)	
Scleroderma pattern, n (%)	7 (17.1)	0 (0)	

Bold indicates statistically significant p values (<0.05).

Mm: millimeter; SD: standard deviation; µm: micrometer.

^a^paired t-test ^b^Wilcoxon signed rank test.

^c^nailfold capillary abnormalities which are also described in adult-onset SLE (4).

### Qualitative capillary patterns

Compared to healthy controls, cSLE-patients showed significantly more abnormal capillary patterns (Z= -5.291, p < 0.001) (Table 2). In total, thirty-two patients (32/41, 78%) showed a specific combination of ‘pericapillary extravasations’ and ‘abnormal capillary shapes’ combined in the same finger (as shown in figure 2), including all patients (n = 7) with a scleroderma pattern. Looking at frequency and localization of these two combined capillary abnormalities, three patients (3/32, 9.4%) showed this combination of capillary abnormalities in all eight examined fingers, 59.4% (19/32) in four or more fingers and 78.1% (25/32) in three or more fingers. Three other patients with high number of ‘pericapillary extravasations’ (with a total count of 96, 71 and 128 extravasations) were also qualitatively analyzed as ‘microangiopathy’, but these three patients did not show the specific combination with ‘abnormal capillary shapes’.

Clinical details of cSLE-patients with capillary scleroderma pattern (n = 7/41, 17.1%) are shown in supplementary file 1. Five out of seven patients (71.4%) with a scleroderma pattern had positive anti-RNP antibodies versus 32.4% (n = 11/34) of patients without a scleroderma pattern, this difference was not significant (p = 0.089). None of these patients showed any signs of sclerodactyly nor other classification criteria for SSc. This was also not detected at follow-up (range 1-9 years). Two healthy controls showed one giant capillary (per person) with diameters of 54.7 and 62.4 µm. As no other capillary abnormalities were found in these healthy individuals, this was not scored as a scleroderma pattern in these two healthy controls. Both giants were observed in the second fingers (with more frequent use and risk for trauma) while the giants in cSLE-patients were observed in the fourth/fifth fingers.

### Correlations with demographic and clinical variables

### Capillary morphology

In cSLE-patients, the amount of ‘abnormal shapes per mm’ was significantly correlated with periungual trauma (p = 0.049) and treatment-naivety (p = 0.022). In healthy controls, no correlations were found for the amount of abnormal shapes per mm (supplementary file 2).

### Apical diameter

cSLE patients showed no significant correlation between the presence of giants and Raynaud’s phenomenon (Odds rato (OR) 2.3, 95% confidence interval (CI) 0.48 – 11.08, p = 0.299). There was also no significant correlation between the amount of ‘giants per mm’ and presence of anti-RNP antibodies (supplementary file 3).

### Large pathological hemorrhages

In cSLE-patients, ‘large pathological hemorrhages per mm’ showed a significant correlation with SLEDAI scores (at diagnosis (p = 0.009) and at capillaroscopy (p = 0.002)) and nephritis (p = 0.012). In healthy controls, the amount of ‘large pathological hemorrhages per mm’ was significantly correlated with periungual trauma (p = 0.004) (see Table 3).

**Table 3.** Correlations between clinical and demographical variables and amount of “large hemorrhages per mm”.

Variable	Regression coefficient β (95% CI) cSLE-patients	p-Value	Regression coefficient β (95% CI) healthy controls	p-Value
Skin pigmentation (ordinal)	0.103 (–0.001 – 0.207)	0.052	0.000 (–0.051 – 0.052)	0.990
Trauma	–0.068 (–0.376 – 0.239)	0.656	0.163 (0.054 – 0.271)	**0.004**
Raynaud/acrocyanosis	–0.087 (–0.298 – 0.124)	0.410	–0.037 (–0.247 – 0.174)	0.726
Treatment-naivety	–0.061 (–0.264 – 0.141)	0.543		
Disease duration	–0.002 (–0.006 – 0.001)	0.241		
SLEDAI at diagnosis	0.019 (0.005 – 0.032)	**0.009**		
SLEDAI at capillaroscopy	0.021 (0.008 – 0.035)	**0.002**		
Anti-RNP	–0.017 (–0.224 – 0.189)	0.866		
Cutaneous involvement	–0.128 (–0.337 – 0.081)	0.222		
Neuropsychiatric involvement	0.121 (–0.162 – 0.404)	0.392		
Nephritis	0.260 (0.06 – 0.460)	**0.012**		
Antiphospholipid antibodies	0.000 (–0.071 – 0.072)	0.989		

Bold indicates statistically significant p values (<0.05).

### Pericapillary extravasations

In both cSLE-patients (p < 0.001) and in healthy controls (p = 0.001), the amount of ‘pericapillary extravasations per mm’, was significant positively correlated with darker skin pigmentation (see Table 4). In healthy controls, the presence of ‘pericapillary extravasations’ (observed or not) was significantly correlated with darker skin pigmentation (logistic regression, OR 14.33, 95% CI 3.30-62.32, p < 0.001).

**Table 4.** Correlations between clinical and demographical variables and amount of “pericapillary extravasations per mm”.

Variable	Regression coefficient β (95% CI) cSLE-patients	p-Value	Regression coefficient β (95% CI) healthy controls	p-Value
Skin pigmentation (ordinal)	0.846 (0.536 – 1.157)	**<0.001**	0.146 (0.063 – 0.230)	**0.001**
Trauma	–0.587 (–1.740 – 0.567)	0.310	–0.028 (–0.253 – 0.197)	0.803
Raynaud/acrocyanosis	0.193 (–0.612 – 0.997)	0.631	–0.129 (–0.521 – 0.262)	0.507
Treatment-naivety	0.265 (–0.501 – 1.031)	0.489		
Disease duration	0.011 (–0.002 – 0.024)	0.109		
SLEDAI at diagnosis	0.034 (–0.021 – 0.090)	0.216		
SLEDAI at capillaroscopy	0.025 (–0.31 – 0.081)	0.372		
Anti-RNP	0.518 (–0.249 – 1.284)	0.180		
Cutaneous involvement	–0.423 (–1.218 – 0.373)	0.289		
Neuropsychiatric involvement	–0.855 (–1.901 – 0.192)	0.106		
Nephritis	0.564 (–0.237 – 1.366)	0.163		
Antiphospholipid antibodies	–0.011 (–0.283 – 0.261)	0.934		

Bold indicates statistically significant p values (<0.05).

## Discussion

Our observations confirm that giants, abnormal capillary morphology and capillary hemorrhages are also observed in cSLE, as was already known for adults with SLE.^4^ The uniqueness of our cohort is that more than half of the patients (23/41, 56.1%) were treatment-naive at the moment of capillaroscopy examination. This is the first study to describe abnormal capillary morphology in cSLE since the new published definitions for abnormal capillary shapes from EULAR SG MCRD in 2016.^6^ In this cross-sectional study and compared to healthy controls, cSLE-patients show significantly more giant capillaries, abnormal capillary morphology and capillary hemorrhages, both in absolute numbers (per mm) as well as in percentage of patients. The high number (median 1.1 per mm) of capillary hemorrhages in cSLE patients and the observation of two different subtypes of capillary hemorrhages are the other prominent findings of our study. Large hemorrhages were also observed in healthy controls but these were significantly correlated with trauma, which seems a logical explanation.

We found a significant correlation between the amount of large hemorrhages and SLEDAI score (at diagnosis and at capillaroscopy). Significantly higher SLEDAI scores in adult SLE with major capillary changes (defined by abnormal shapes and capillary hemorrhages) have been described before, further specified by a correlation between more capillary hemorrhages in the patient group with a SLEDAI score of >12.^27^ Ingegnoli also showed a linear correlation with SLEDAI score and severity of capillary abnormalities, by semi-quantitatively scoring patterns between 0-2.^28^ Approximately half of patients (56%) in our cohort were analyzed at the moment of diagnosis (treatment naïve). Improvement of abnormal capillary changes due to therapeutic intervention has been described in SSc.^29,30^ In our cohort, the median SLEDAI score of 5 at the moment of capillaroscopy is interpreted as a low disease activity score which may underestimate our results. Our significant correlation between the amount of abnormal capillary shapes and treatment-naivety confirms this. Presence of nephritis was significantly correlated with large pathological hemorrhages, while no other disease manifestations showed correlations with capillary abnormalities. An explanation could be that the found capillary abnormalities are representative for SLE in general and not specific for certain clinical symptoms of this severe disease.

A novel finding in this study was the observation of ‘pericapillary extravasations’: small point-shaped hemorrhages surrounding the capillary apex. These extravasations were observed in significantly higher frequency and count per mm in cSLE-patients, as compared to healthy controls. The ‘pericapillary extravasations’ seem a distinct subtype of capillary hemorrhage and were six times more often observed than ‘large pathological hemorrhages’, when analyzed per mm. Interestingly, pericapillary extravasations were not correlated with periungual trauma (Table 4), suggesting a pathophysiological origin such as endothelial wall damage. To our knowledge, such extravasations have been sporadically described in adult SLE-patients (and never in children), as “pearl necklaces of extravasates” or “extravasations of red blood cells, with the impression of punched out windows”.^31,32^ A possible explanation for this new observation could be that the quality and resolution of images from NVC have significantly improved in the last years. Hypothetically, this subtype of capillary hemorrhages might be small extravasations from a vulnerable capillary possibly due to endothelial activation and damage. It is possible that these ‘pericapillary extravasations’ are a reflection of endothelial dysregulation, as has been demonstrated in SLE-patients,^33–35^ leading to vasculopathy, which may be related to the pathogenesis of SLE. Pericapillary extravasations do not show migration towards the peripheral area (along with nail growth) as large hemorrhages do in a scleroderma pattern. Possibly, smaller hemosiderin deposits are cleared faster by phagocytic cells. The endothelial activation and damage, as described in SSc,^36^ does also seem to play a role in SLE.^33–35^ SLE occurs 2 to 4 times more frequently among non-white populations^37^ and this non-white population also seems to have a more severe disease course.^11,38^ It might be that the significant higher amount of extravasations, found in our non-white cSLE-patients, reflects this.

The combination of ‘pericapillary extravasations and abnormal capillary shapes’ were mostly observed in the fourth and fifth digits. These digits are less used in daily activities, suggesting that these capillary changes are less likely to be caused by trauma and further supporting a possible origin from a pathophysiological damage of endothelium. Multivariate analysis to determine correlations between detection of such ‘specific microangiopathy pattern’ with disease characteristics could not be performed due to small sample size of the group (< 10 subjects) that did not show this ‘specific microangiopathy pattern’ (n = 9/41).^39^ All seven cSLE-patients with a scleroderma pattern also showed this specific combination of ‘capillary abnormal shapes and pericapillary extravasations’. However, these patients did not have any other clinical criteria for SSc and positive anti-RNP antibodies were not significantly more detectable in these patients. Longitudinal studies are needed for clinical follow-up of these cSLE-patients with a capillary scleroderma pattern for a correct interpretation of this finding. The hypothesis for pathogenesis of a scleroderma pattern is that the capillary first typically enlarges due to endothelial damage forming a micro-aneurysmatic giant capillary, which might subsequently lead to a capillary microhemorrhage. These microhemorrhages are closely associated with the enlarged loops and have an obvious apical capillary genesis.^1^ In our cohort we did not observe a correlation between the amount of large hemorrhages and the number of giant capillaries per mm (regression coefficient β 0.71, 95% CI -0.06 – 1.49, p = 0.07). This observation also leads to the question if the pathogenesis of large capillary hemorrhages is different in SLE than in scleroderma, both distinct systemic autoimmune diseases with incidentally clinical overlap with other connective tissue diseases.

The limitations of this study include the relatively small sample size of this cSLE-cohort with 41 patients, due to the rarity of this disease. Our male/female ratio was 1/7, representing the general known male/female ratio of 1/8-10 in adults with SLE^37^ and 1/5-6 in cSLE.^14^ Secondly, it is known that SLE occurs 2 to 4 times more frequently among non-white populations^37^ which is also shown in our data (63.4% non-white). In cSLE, a median onset of 12.6 years (IQR 10.4-14.5) at diagnosis is described in the literature^14^ which corresponds with our cohort with a median age of 14 years (IQR 12.5-16) at diagnosis. Although median age was significantly lower in the healthy cohort (12 versus 17 years), this difference will probably not make a difference for our outcome data, as it still concerned a pediatric (teenage) population. The same argument applies to matching of gender which was significantly different but with percentages of 87 versus 70% a majority of females in both cohorts.

This study confirms that children with SLE, like adult SLE-patients, also show significantly more giants, abnormal capillary morphology and capillary hemorrhages, when compared to healthy controls. In our study, these abnormal capillary findings were significantly correlated with SLEDAI scores, treatment-naivety and nephritis, thus making nailfold capillary abnormalities potentially interesting as disease biomarker(s). A prominent finding was the observation of a newly described subtype of capillary hemorrhage which we called “pericapillary extravasations”. By assessment of intra- and inter-observer variability we need to determine if these pericapillary extravasations are reproducible, to confirm if they are a distinct finding from large capillary hemorrhages.

## Supplemental Material

sj-pdf-1-lup-10.1177_0961203321998750 - Supplemental material for Nailfold capillary abnormalities in childhood-onset systemic lupus erythematosus: a cross-sectional study compared with healthy controls Click here for additional data file. Supplemental material, sj-pdf-1-lup-10.1177_0961203321998750 for Nailfold capillary abnormalities in childhood-onset systemic lupus erythematosus: a cross-sectional study compared with healthy controls by Dieneke Schonenberg-Meinema, Sandy C Bergkamp, Amara Nassar-Sheikh Rashid, Leontien B van der Aa, Godelieve J de Bree, Rebecca ten Cate, Maurizio Cutolo, A Elisabeth Hak, Petra CE Hissink Muller, Marieke van Onna, Taco W Kuijpers, Vanessa Smith and J Merlijn van den Berg in Lupus

sj-pdf-2-lup-10.1177_0961203321998750 - Supplemental material for Nailfold capillary abnormalities in childhood-onset systemic lupus erythematosus: a cross-sectional study compared with healthy controls Click here for additional data file. Supplemental material, sj-pdf-2-lup-10.1177_0961203321998750 for Nailfold capillary abnormalities in childhood-onset systemic lupus erythematosus: a cross-sectional study compared with healthy controls by Dieneke Schonenberg-Meinema, Sandy C Bergkamp, Amara Nassar-Sheikh Rashid, Leontien B van der Aa, Godelieve J de Bree, Rebecca ten Cate, Maurizio Cutolo, A Elisabeth Hak, Petra CE Hissink Muller, Marieke van Onna, Taco W Kuijpers, Vanessa Smith and J Merlijn van den Berg in Lupus

sj-pdf-3-lup-10.1177_0961203321998750 - Supplemental material for Nailfold capillary abnormalities in childhood-onset systemic lupus erythematosus: a cross-sectional study compared with healthy controls Click here for additional data file. Supplemental material, sj-pdf-3-lup-10.1177_0961203321998750 for Nailfold capillary abnormalities in childhood-onset systemic lupus erythematosus: a cross-sectional study compared with healthy controls by Dieneke Schonenberg-Meinema, Sandy C Bergkamp, Amara Nassar-Sheikh Rashid, Leontien B van der Aa, Godelieve J de Bree, Rebecca ten Cate, Maurizio Cutolo, A Elisabeth Hak, Petra CE Hissink Muller, Marieke van Onna, Taco W Kuijpers, Vanessa Smith and J Merlijn van den Berg in Lupus
