# Supplementary material for: Nailfold capillary abnormalities in childhood-onset systemic lupus erythematosus: a cross-sectional study compared with healthy controls
Source: Lupus. 2021 Mar 3;30(5):818–27. doi: 10.1177/0961203321998750 (PMC8020305; doi:10.1177/0961203321998750)
Supplement: sj-pdf-1-lup-10.1177_0961203321998750 - Supplemental material for Nailfold capillary abnormalities in childhood-onset systemic lupus erythematosus: a cross-sectional study compared with healthy controls [file sj-pdf-1-lup-10.1177_0961203321998750.pdf]

Supplementary file 3. Correlations between clinical and demographical variables and amount of "giant capillaries per mm"

Variable	Regression coefficient β (95% CI) cSLE	p-value
Skin pigmentation (ordinal)	-0.013 (-0.057 – 0.030)	0.543
Trauma	-0.026 (-0.149 – 0.098)	0.678
Raynaud/acrocyanosis	0.054 (-0.030 – 0.137)	0.200
Treatment-naivety	0.013 (-0.068 – 0.095)	0.740
Disease duration	-0.001 (-0.002 – 0.000)	0.166
SLEDAI at diagnosis	-0.002 (-0.008 – 0.004)	0.487
SLEDAI at capillaroscopy	0.001 (-0.005 – 0.007)	0.629
Anti-RNP	0.052 (-0.029 – 0.134)	0.200
Cutaneous involvement	-0.022 (-0.107 – 0.063)	0.598
Neuropsychiatric involvement	-0.028 (-0.142 – 0.086)	0.619
Nephritis	-0.034 (-0.120 – 0.052)	0.430
Antiphospholipid antibodies	-0.002 (-0.031 – 0.026)	0.874

Bold indicates statistically significant p values (<0.05)
