# Supplementary material for: Nailfold capillary abnormalities in childhood-onset systemic lupus erythematosus: a cross-sectional study compared with healthy controls
Source: Lupus. 2021 Mar 3;30(5):818–27. doi: 10.1177/0961203321998750 (PMC8020305; doi:10.1177/0961203321998750)
Supplement: sj-pdf-2-lup-10.1177_0961203321998750 - Supplemental material for Nailfold capillary abnormalities in childhood-onset systemic lupus erythematosus: a cross-sectional study compared with healthy controls [file sj-pdf-2-lup-10.1177_0961203321998750.pdf]

Supplementary file 2. Correlations between clinical and demographical variables and amount of “abnormal shapes per mm”

**Variable**	**Regression coefficient β (95% CI) cSLE**	**p-value**	**Regression coefficient B (95% CI) healthy controls**	**p-value**
Skin pigmentation (ordinal)	0.037 (-0.1 – 0.173)	0.587	0.021 (-0.035 – 0.078)	0.454
Trauma	0.369 (0.001 – 0.737)	**0.049**	-0.012 (-0.146 – 0.121)	0.854
Raynaud/acrocyanosis	0.184 (-0.076 – 0.444)	0.160	-0.066 (-0.298 – 0.167)	0.572
Treatment-naivety	0.281 (0.042 – 0.519)	0.022		
Disease duration	0.005 (0.001 – 0.01)	**0.01**		
SLEDAI at diagnosis	0.009 (-0.009 – 0.028)	0.324		
SLEDAI at capillaroscopy	-0.01 (-0.029 – 0.008)	0.257		
Anti-RNP	0.226 (-0.023 – 0.475)	0.074		
Cutaneous involvement	-0.145 (-0.408 – 0.118)	0.271		
Neuropsychiatric involvement	0.065 (-0.293 – 0.422)	0.716		
Nephritis	-0.137 (-0.405 – 0.132)	0.309		
Antiphospholipid antibodies	-0.029 (-0.118 – 0.061)	0.518		

Bold indicates statistically significant p values (<0.05)
