# Supplementary material for: Nailfold capillary abnormalities in childhood-onset systemic lupus erythematosus: a cross-sectional study compared with healthy controls
Source: Lupus. 2021 Mar 3;30(5):818–27. doi: 10.1177/0961203321998750 (PMC8020305; doi:10.1177/0961203321998750)
Supplement: sj-pdf-3-lup-10.1177_0961203321998750 - Supplemental material for Nailfold capillary abnormalities in childhood-onset systemic lupus erythematosus: a cross-sectional study compared with healthy controls [file sj-pdf-3-lup-10.1177_0961203321998750.pdf]

**Supplementary file 1.** Clinical characteristics of cSLE-cases with capillary scleroderma pattern, n=7.

| | **Case 1** | **Case 2** | **Case 3** | **Case 4** | **Case 5** | **Case 6** | **Case 7** |
|---|---|---|---|---|---|---|---|
| Demographics (age at presentation) | Girl, Caucasian, 12 years old | Girl, Caucasian, 13 years old | Boy, Asian, 13 years old | Girl, Afro-Caribbean, 15 years old | Girl, Afro-Caribbean 17 years old | Girl, Afro-Caribbean | Girl, Caucasian, 17 years old |
| **Clinical symptoms** | Butterfly rash of erosive ulcerative skin disease, extensive oral aphtous ulcers, chilblains, auto-immune hepatitis, splenomegaly with calcifications, leukopenia, thrombocytopenia | Butterfly rash and discoid skin lesions trunk, arms after sun exposure, apthous ulcers, leukopenia | Pleuritis, pericarditis, ascites, myositis, polyarthritis, fever, lymphadenopathy, deep venous thrombosis, hepatosplenomegaly, aphtous ulcers, leukopenia | Pleuritis, pericarditis, myositis, polyarthritis, fever, aphtous ulcers, leukopenia, thrombocytopenia | Aphtous ulcers, lymphadenopathy, skin rash (resulting in hyperpigmentation) | Vasculitis, arthritis, nephritis, mood disorder (intracranial hypertension/resorption) | Aphtous ulcers, leukopenia, thrombocytopenia |
| **Skin biopsy** | Vacuolar degeneration of the basal layer with abundant nuclear dust, also localized around the superficial blood vessels.Immunofluorescence: positive lupus band with (granular) staining of IgG, IgM, C1q and C3 | The epidermis shows a hyperkeratotic basket-weave stratum corneum, with some vacuolar degeneration of the basal layer. The epidermis is atrophic with follicular plugging. Below the epidermis a mild perivascular and perifollicular, predominantly lymphocytic infiltrate | - | - | A-specific inflammation lymph node, lip biopsy inconclusive | Around superficial vessels, subcutaneous tissue and deep dermal plexus inflammation with lymphocytes, histiocytes, neutrophilic granulocytes en abundant nuclear dust. Also eosinophilic granulocytes around vessels and focal interstitial degranulation. Vessel wall is swollen with focal fibroid change. Immunofluorescence: depositions of complement factors (C1q>>C3c), combined with IgM (and some IgG) | - |
| **ANA / anti-ds-DNA** | positive / positive | positive / positive | positive / positive | positive / positive | positive / negative | positive / positive | positive / positive |
| **Other auto-antibodies** | anti-RNP, anti-Ro52, anti-SS-A, anti-Sm | anti-RNP, anti-SS-A, anti-Sm | anti-RNP, anti-SS-A, anti-Sm, rheumatoid factor | anti-RNP, anti-Sm, anti-Ro52, anti-ds-DNA, rheumatoid factor | none | anti-C1q antibodies | Anti-RNP, anti-Sm |
| **Anti-phospholipid antibodies** | negative | negative | positive | negative | positive | negative | negative |
| **C3/C4** | low | low | low | low | normal | low | normal |
| **Coombs test** | positive | positive | positive | positive | positive | positive | negative |
| **SLEDAI at presentation** | 17 | 10 | 10 | 29 | 4 | 35 | 4 |
| **SLEDAI at capillaroscopy** | 17 | 10 | 8 | 6 | 4 | 35 | 4 |
| **Disease duration at capillaroscopy** | at diagnosis | at diagnosis | 4 years | 5 years | at diagnosis | at diagnosis | at diagnosis |
| **Discoloration of fingers** | acrocyanosis in winter | no | biphasic Raynaud's phenomenon | acrocyanosis during whole year | no | no | Raynaud |
| **Sclerodactyly** | no | no | no | no | no | no | no |
| **Pulmonary disease** | no | no | restrictive pulmonary function | no | no | no | no |
| **Nephritis** | no | no | proteinuria: biopsy refused by parents/patient | nephritis class V | no | nephritis class IV | no |
| **Medication at capillaroscopy (and ever used)** | None (prednisolone, hydroxychloroquine azathioprine, rituximab, mycophenolate mofetil, belimumab) | None (prednisolone, hydroxychloroquine azathioprine) | prednisolone, hydroxychloroquine, methotrexate (rituximab, mycophenolate mofetil) | prednisolone, hydroxychloroquine, mycophenolate mofetil, cyclophosphamide (azathioprine, methotrexate, rituximab, belimumab,) | Prednisolone (hydroxychloroquine) | None (prednisolone, hydroxychloroquine, mycophenolate mofetil) | (hydroxychloroquine, nifedipine, prednisolone) |
| **Follow-up period** | 4 years | 5 years | 8 years | 9 years | Lost to follow-up | 2 years | 1 year |
